# Shifting epidemiological patterns and strain replacement of hand, foot, and mouth disease in southern China, 2009–2022: A longitudinal study

**DOI:** 10.1371/journal.pone.0341302

**Published:** 2026-01-22

**Authors:** Yanan Hou, Huafeng Chen, Yi Jiang, Hao Huang, Zhenlian Tan, Eric J. Nehl, Hai Li, Jiangshan Wang, Jinda He, Chen Zhong, Yuqi Wang, Jian Xiao, Minmei Chen, Zhigang Zheng

**Affiliations:** 1 Department of Epidemiology, School of Public Health and Management, Guangxi University of Chinese Medicine, Nanning, Guangxi, China; 2 Institute of Acute Infectious Diseases Control and Prevention, Guangxi Zhuang Autonomous Region Center for Disease Prevention and Control, Nanning, Guangxi, China; 3 Bureau of Disease Control and Prevention, Wuzhou, Guangxi, China; 4 Division of Infectious Diseases Control and Prevention, Wuzhou City Center for Disease Prevention and Control, Wuzhou, Guangxi, China; 5 Department of Behavioral Sciences and Health Education, Rollins School of Public Health, Emory University, Atlanta, GeorgiaUnited States of America; 6 Guangxi Key Laboratory of Translational Medicine for Treating High-Incidence Infectious Diseases with Integrative Medicine, Nanning, Guangxi, China; 7 University Engineering Research Center of Characteristic Traditional Chinese Medicine and Ethnomedicine, Nanning, Guangxi, China; 8 Guangxi Engineering Research Center for High-Value Utilization of Guangxi-Produced Authentic Medicinal Herbs, Nanning, Guangxi, China; Institut Pasteur, FRANCE

## Abstract

**Background:**

China launched a universal campaign of monovalent-inactivated EV-A71 vaccine in 2016. However, the monovalent-inactivated vaccine is ineffective against HFMD with other etiologies. To date, little is known about the epidemiological patterns and the replacement of the predominant pathogens that induce HFMD after the universal vaccination campaign.

**Methods:**

Using HFMD surveillance and VP1 sequencing data in pre- and post-vaccination periods, time series analysis, and Wavelet approaches, we identified pathogen replacement, changes in the viral spectrum and epidemiological patterns of HFMD.

**Results:**

A prolonged but lower peak of HFMD occurred in the spring, and a late, higher peak presented during fall in the post-vaccination period. The typical half-year transmission cycle disappeared, the one-year cycle did not change, and the CVA16 and CVA6 serotypes have the potential to replace EV-A71 as the predominant strains.

**Conclusions:**

The epidemic pattern of HFMD has shifted after the vaccination campaign. While the EV-A71 vaccine reduced transmission in the spring season, it had little impact on reducing cases in fall. Meanwhile, potential new predominant strains have emerged. Public health system should incorporate enhanced surveillance by including whole genome sequencing to ensure close monitoring of serotype replacement and to assess the need for multivalent vaccines across Southern China.

## Introduction

Hand, foot, and mouth disease (HFMD) is a common infectious disease with blistering, erosion, or Vesiculopapular rash in the mouth, body, and limbs of the infected individual [1]. HFMD predominantly infects children under five years of age. Most HFMD cases present with mild symptoms. Severe neurological complications such aseptic meningitis (AM), brain stem encephalitis (BE), and acute flaccid myelitis (AFM) have been reported in multiple countries [2–8]. A global pandemic of HFMD caused by the EV-A71 serotype emerged in the late 1980s. Subsequent epidemics, often associated with severe neurological complications, were reported in the Hong Kong Special Administrative Region, Brazil, Japan, Australia, the United States, the Netherlands, and other countries [9–13]. The pandemic of HFMD caused high disease burden worldwide since then. HFMD was estimated to cause 97,000 disabilities adjusted life year (DALY) losses per annum across Asia [14]. In South Korea, HFMD epidemics recur between April and August, and the annual economic burden is estimated to be US $100 million [15]. Outbreaks of HFMD in China resulted in millions of children being infected, hundreds of thousands of these cases were severe, and thousands of children died [16–21]. The mean quality adjusted life year (QALY) losses per HFMD episode for mild outpatients, mild inpatients and severe cases were 3.6 (95%CI 3.4, 3,9), 6.9 (95%CI 6.4, 7.4) and 13.7 (95%CI 12.9, 14.5) per 1000 persons [22]. There is no specific antiviral treatment for HFMD, severe cases can progress to fatal outcomes, further highlighting the urgency of disease preventing and control.

Enteroviruses (EVs) are the major pathogens that cause HFMD and belong to the Family *Picornaviridae* including EV-A71, the family of Coxsackieviruses (CV), such as CVA2, CVA4, CVA6, CVA10, and CVA16, and the family of Echoviruses (E), such as E3, E6 and E9. EV-A71 and CVA16 [23,24]. The dominant pathogens related to HFMD in China, EV-A71, CVA16, and CVA6, belong to the species Enterovirus A (EV-A), genus Enterovirus in the Family *Picornaviridae*. These viruses are small, non-enveloped with icosahedral symmetry, and contain a positive sense, single-stranded RNA genome. The complete genome is approximately 7.4 kb in length. The genome coding sequence is flanked with a 5′ untranslated region (5′UTR) linking to the virus-encoded peptide (VPg) and a poly-A tail at the 3′ untranslated region (3′UTR). The genome contains one open reading frame (ORF) with 3 regions: P1, P2, and P3. P1 encodes VP1–4 structural proteins, P2 encodes 2A, 2B, and 2C non-structural proteins, and P3 encodes 4 non-structural proteins: 3A-3D [25–27]. Based on the sequence of VP1 region, EV-A71 viruses are classified into eight genotypes (A–H) [28,29]. The prototype of genotype A, BrCr, was identified first in a patient with encephalitis in California, USA, in 1969 [6]. Genotypes B and C comprised multiple sub-genotypes, B0–B5 and C1–C6 [23,30]. Genotypes D and G were discovered in India from patients with acute flaccid paralysis, and genotypes E and F were discovered in Africa and Madagascar, respectively [3]. Genotype H was detected in sewage samples in Pakistan [4]. Similarly, CVA16 comprises four genotypes (A–D) based on VP1 or VP4 sequences [2,9–12]. Prototype G-10 is the only member of genotype A, while genotype B is further divided into sub-genotypes B1 (B1a, B1b, and B1c), B2 [2,9], and B3 [31]. CVA16 genotype C was discovered in Peru [32], while genotype D was discovered in Peru, France, and China [33,34]. CVA6 can be categorized into 4 genotypes (A–D). The prototype strain Gdula isolated in the USA in 1949 is classified as genotype A. Genotypes B, C, and D, are subdivided into sub-genotypes B1–B2, C1–C2, and D1–3, respectively [35,36].

Herd immunity through vaccination is considered the most effective means to reduce the disease burden of HFMD. A universal vaccination campaign using the inactivated monovalent EV-A71 vaccine was initiated in China at the end of 2016. Clinical trials have confirmed that this vaccine effectively protects 95–97% of individuals against EV-A71 infection, significantly reducing the burden of EV-A71-related HFMD. However, it provides no cross-protection against infections caused by other enteroviruses such as CVA16, creating conditions for potential shifts in the predominant pathogen spectrum of HFMD [25]. In addition, EVs are RNA viruses that frequently mutate due to lack of RNA-dependent RNA polymerase proofreading activity during genome replication. Therefore, it is hypothesized that the inactivated monovalent EV-A71 vaccine may not reduce the reported cases of HFMD significantly and may promote changes in the pathogen spectrum.

Researchers across China have confirmed that the epidemiology of HFMD has changed due to the impact of the EV-A71 vaccination [31,32,37]. Research in Guangxi has found that the incidence rate associated with EV-A71 and case severity rate decreased after the vaccination campaign [33]. However, the majority of studies focused on the changing of epidemiological profiles of HFMD with limited examination of pathogen types. This study investigated the changing epidemiological patterns and predominant strain replacement of HFMD in Southern China with combining epidemiological data and pathogen spectrum analysis. Therefore, the aim of this study is to inform a robust vaccination or alternative strategy for HFMD control in Southern China in the context of the universal EV-A71 vaccination campaign.

## Materials and methods

### Participants

Weekly reports of HFMD incidence between 1 January 2009 and 31 December 2022 from Guangxi were obtained from a national surveillance system maintained by the Chinese Center for Disease Control and Prevention (China CDC) in Beijing, China. HFMD diagnosis was based on laboratory-based pathogen detection, clinical symptoms, and the epidemiological links to laboratory-confirmed cases. HFMD diagnosis followed the Chinese Guidelines for the Diagnosis and Treatment of Hand, Foot and Mouth Disease (2010 Version) [38].

Clinical symptoms for HFMD included papules, or vesicular rashes on hands, feet, mouth or buttocks, with or without fever. Cases were confirmed through throat swab or nasal swab (targeting EV-A71, CVA16, CVA10, CVA6 and other serotypes) via RT-PCR, quantitative RT-PCR, or positive fecal culture. We provide the sample sizes including the number of cases for epidemic pattern analysis, clinical specimens for cases confirmation, and VP1 sequences for strain replacement analysis.

### Specimen collection

A total of 85,702 clinical specimens including throat swabs, nasal swabs, and fecal samples were collected during the study period. The weight of the collected fecal specimens was 5–8 g/sample. Specimens were stored in sterile tubes immediately after collection and labeled with a unique ID number. Specimens were then transported at 0–4°C to the provincial laboratory and stored at −80°C. In total, 75,477 specimens were confirmed as EV positive.

### Viral RNA extraction and sequencing

A total of 75,477 specimens with positive viral isolation were used for RNA extraction. Viral RNA from throat swabs, or nasal swabs, or fecal samples was extracted using the Tianlong Viral RNA Extraction Kit (Tianlong, Xi’an xi, China) based upon instructions of the manufacturer. Similarly, Viral RNA was extracted from 200 μL of supernatant using the viral RNA mini kit (Qiagen, Hilden, Germany) according to manufacturer instructions. Finally, RNA from each sample was examined using real-time RT-PCR kits (Diagnostic Kit for human enteroviruses, EV-A71, and CVA16, Jiangshu Shuoshi Biological Technology Co., Ltd, Taizhou, China) following manufacturer instructions Patients who tested positive were classified as having EV-A71, CVA6, CVA10, or CVA16 infections. Specimens with a Ct value > 38 or with no detectable pathogens were confirmed as negative. Specimens with a sigmoidal amplification curve and a Ct value =< 35 were confirmed to be positive. For specimens where the amplification curve was sigmoidal and a Ct value was between 35 and 38, the test was repeated. Samples were confirmed positive if the amplification curve remained sigmoidal within this Ct range. Amplified products were sent to Sangon Biotech Co., Ltd (Shanghai, China) for DNA Sanger sequencing.

### Datasets of EV-A71, CVA16 and CVA6 VP1 nucleotide sequences

The assembled complete VP1 sequences were compared using the MEGA X [39] program. Phylogenetic trees were constructed using the neighbor-joining method with Figtree [40]. Branch lengths were determined by the maximum-likelihood method. The reliability of the neighbor-joining tree was estimated by bootstrap analysis with 1,000 pseudo replicate data sets. Previously sequenced EV-A71 strains BrCr-CA-70 and the standard sequences of sub-genotype of EV-A71, CVA6, CVA10, and CVA16 from NCBI GenBank database were also included in the analysis. In total, 647 VP1 gene sequences were examined.

### Estimation of genetic distance and evolution

There are eleven genotypes of EV-A71, including A, B1, B2, B3, B4, B5, C1, C2, C3, C4, and C5. Reference VP1 sequences of each genotype were downloaded from GenBank and compared with the sequences isolated from our samples to identify the predominant circulating strains. Virus strains of the same sub-genotype typically share over 75% nucleotide similarity and more than 85% amino acid similarity. VP1 nucleotide sequences were aligned using Muscle in AliView v1.26 (https://ormbunkar.se/aliview/). A maximum likelihood (ML) phylogenetic tree was estimated using IQ-TREE (v.1.6.12) under the best-fit nucleotide substitution model (TIM3e+R2 for EV-A71, TNe + I for CVA16 and TNe + G4 for CVA6) determined by Model Finder. The bootstrap with 1,000 replicates was applied.

### Definitions

1. Pre- and post-vaccination period: A universal campaign of the monovalent EV-A71 vaccine was introduced at the end of 2016 in mainland China. This study defined 2009–2016 as the pre-vaccination period, and 2017–2022 as the post-vaccination period.
2. Major and minor epidemic year: Following a minor outbreak in one year, a larger outbreak typically occurred the following year. We defined the previous year with the smaller outbreak as the minor epidemic year, and the subsequent year with the larger outbreak as the major epidemic year. In this study, minor epidemic years consistently occurred in odd-number years, while the major epidemics occurred in even-number years.

### Statistical analysis

Time series methods were used to analyze the changing seasonal patterns of HFMD from 2009 to 2022. Descriptive statistics were used to compare epidemiological peaks and their timing before and after the vaccination campaign. Wavelet Power analysis was used to identify epidemiological periodicity with the Rwavelet package [41]. By combining epidemiological data and VP1 sequencing data, we identified the replacement of predominant circulating strain. Data were analyzed using R-software version 4.2.3 (http://cran.r-project.org)). *P* < 0.05 indicated statistically significant differences.

## Results

### Demographic characteristics of EV positive individuals

A total of 75,477 cases were confirmed as EV positive in the study period. Out of the confirmed cases, 47,668 (63.16%) were male, 27,809 (36.84%) were infants aged under one year, 13,675(18.12%) were severe cases. There were 410 (0.54%) fatal cases. A total of six enterovirus serotypes were detected in this study, including EV-A71, CVA16, CVA6, CVA10, as well as two subgroups of “other enteroviruses” (such as Echoviruses E3 [E3, EV-B], echovirus 6 [E6, EV-B], non-target Coxsackievirus CVA2 [EV-A], and CVA4 [EV-A], see Table 1 for the detailed classification). Of the identified serotypes, other EVs was the most prevalence serotype (37.02%, 27,941/75,477), followed by the CVA16 (25.37%, 19,150/75,477) and the EV-A71 (23.33%, 17,607/75,477). Notably, CVA6 and CVA10 serotypes were classified as distinct serotypes only from 2018 and onward, with the overall percentages of 12.15% (9,168/75,477) for CVA6 and 2.13% (1,611/75,477) for CVA10 (Table 1).

**Table 1:** Demographic characteristics of the participants with specimen samples in Guangxi, Southern China (n, %).

Items	2009	2010	2011	2012	2013	2014	2015	2016	2017	2018	2019	2020	2021	2022
Case category														
Mild	906(86.29)	1611(48.48)	3918(92.25)	5003(72.58)	4804(93.46)	5411(67.07)	5115(87.63)	5620(67.98)	5820(67.27)	7200(95.91)	5626(95.13)	2513(98.28)	6037(97.45)	1808(98.96)
Severe	136(12.95)	1606(48.33)	309(7.28)	1791(25.98)	328(6.38)	2549(31.59)	711(12.18)	2616(31.64)	2819(32.58)	304(4.05)	285(4.82)	44(1.72)	158(2.5’5)	19(1.04)
Fatal	8(0.76)	106(3.19)	20(0.47)	99(1.44)	8(0.16)	108(1.34)	11(0.19)	31(0.37)	13(0.15)	3(0.04)	3(0.05)	0(0.00)	0(0.00)	0(0.00)
Total	1050	3323	4247	6893	5140	8068	5837	8267	8652	7507	5914	2557	6195	1827
**Gender**														
Male	682(64.95)	2100(63.20)	2733(64.35)	4433(64.31)	3304(64.28)	5181(64.22)	3670(62.87)	5239(63.37)	5424(62.69)	4613(61.45)	3692(62.43)	1574(61.56)	3888(62.75)	1135(62.12)
Female	368(35.05)	1223(36.80)	1514(35.65)	2460(35.69)	1836(35.72)	2887(35.78)	2167(37.13)	3028(36.63)	3228(37.31)	2894(38.55)	2222(37.57)	983(38.44)	2307(37.25)	692(37.88)
Male: female	1.85:1	1.72:1	1.81:1	1.80:1	1.80:1	1.79:1	1.69:1	1.73:1	1.68:1	1.59:1	1.66:1	1.60:1	1.69:1	1.64:1
**Age Group (Year)**														
~1	593(56.48)	1703(51.25)	1851(43.59)	3270(47.44)	2798(54.44)	4127(51.15)	3151(53.98)	3778(45.70)	4899(56.61)	3200(42.63)	2739(46.31)	999(39.02)	1815(29.30)	591(32.35)
1 ~ 3	355(33.81)	1241(37.35)	1900(44.75)	2789(40.46)	1913(37.22)	3154(39.09)	2190(37.52)	3473(42.01)	2893(33.44)	3080(41.03)	1910(32.30)	1088(42.50)	2606(42.07)	795(43.51)
3 ~ 5	73(6.95)	285(8.58)	394(9.28)	623(9.04)	323(6.28)	631(7.82)	387(6.63)	836(10.11)	680(7.86)	975(12.99)	422(7.14)	363(14.18)	1361(21.97)	327(17.9)
5 ~ 10	29(2.76)	79(2.38)	89(2.10)	185(2.68)	93(1.81)	139(1.72)	98(1.68)	168(2.03)	151(1.75)	199(2.65)	101(1.71)	93(3.63)	391(6.31)	104(5.69)
10~	0(0.00)	15(0.45)	12(0.28)	26(0.38)	13(0.25)	17(0.21)	11(0.19)	12(0.15)	23(0.27)	1(0.01)	0(0.00)	17(0.66)	22(0.36)	10(0.55)
Unknown	0(0.00)	0(0.00)	0(0.00)	0(0.00)	0(0.00)	0(0.00)	0(0.00)	0(0.00)	6(0.07)	52(0.69)	742(12.54)	0(0.00)	0(0.00)	0(0.00)
**Serotype**														
EV-A71	240(22.86)	2176(65.48)	990(23.31)	3985(57.81)	482(9.38)	3419(42.38)	957(16.40)	2823(34.15)	2164(25.01)	242(3.22)	46(0.78)	1(0.03)	50(0.81)	32(1.75)
CVA16	409(38.95)	203(6.11)	2004(47.19)	556(8.07)	1596(31.05)	1577(19.55)	532(9.11)	2682(32.44)	285(3.29)	4366(58.16)	1367(23.11)	420(16.46)	2994(48.33)	159(8.71)
CVA6	0(0.00)	0(0.00)	0(0.00)	0(0.00)	0(0.00)	0(0.00)	0(0.00)	0(0.00)	0(0.00)	651(8.67)	3629(61.36)	1272(49.743)	2514(40.57)	1102(60.32)
CVA10	0(0.00)	0(0.00)	0(0.00)	0(0.00)	0(0.00)	0(0.00)	0(0.00)	0(0.00)	0(0.00)	791(10.54)	15(0.25)	545(21.31)	128(2.07)	132(7.22)
Other Evs	401(38.19)	944(28.41)	1253(29.50)	2352(34.12)	3062(59.57)	3072(38.08)	4348(74.49)	2762(33.41)	6203(71.69)	1457(19.41)	857(14.49)	319(12.47)	509(8.22)	402(22.00)

Note: “other EVs”refers to the serotypes of Echoviruses E3, E6, Coxsackievirus A2, and A4.

### Changing of the seasonal pattern

**Fig 1:**
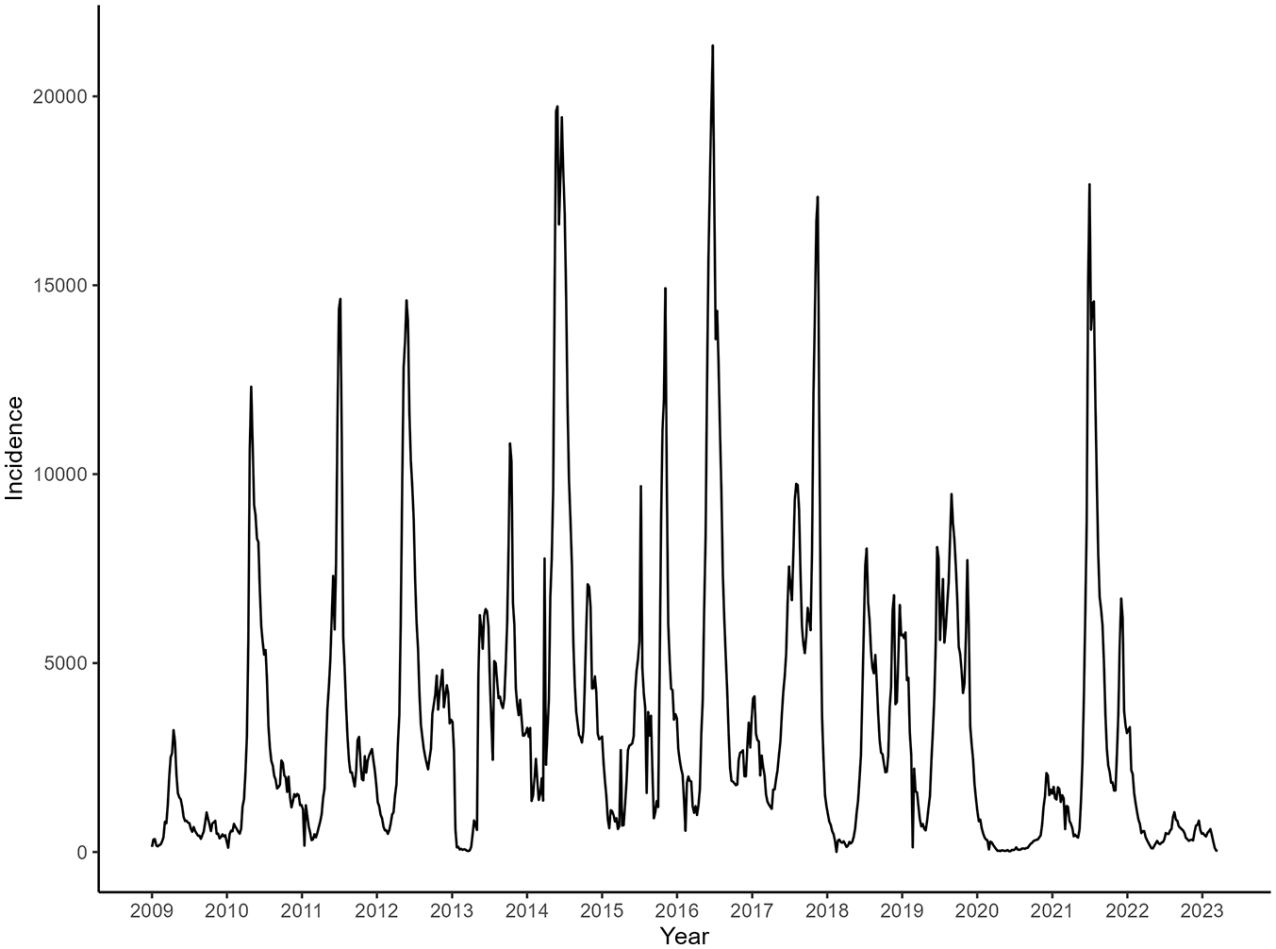
Weekly reported cases incidence of HFMD (Y-axis) with time series approach across 2009–2022 (X-axis). The line chart depicts the annual trend of the incidence rate of HFMD, showing seasonal fluctuations before and after vaccination.

Of the 225,000 (378.10/100,000) reported cases, 17,545 patients (0.78%) experienced severe symptoms such as cardiopulmonary or neurological complications, and 558 deaths were recorded, resulting in a fatality rate of 0.025%. HFMD exhibited a strong seasonality. In the pre-vaccination period, HFMD was characterized by a seasonal, double-peak pattern within a calendar year. The major peak occurred in the spring, occurring in late April or early May, and a minor peak occurred in the fall. Specifically, the epidemic wave began at week 9 (March), reached a major peak at week 16 (late April), and then started to drop after week 20 (June). The minor wave emerged at week 35 (September), peaked at week 39, and then began to decline at week 40 and then persisted at low levels until the following February. In the post-vaccination period, the seasonal pattern changed. Although cases began to increase in week 9 (March), this increase was smaller peak, more gradual, and tapered off at week 28. The major peak in the post-vaccination period shifted to week 39 (October), with more cases reported during the fall season in the post-vaccination period than before the vaccination campaign (Fig 1).

### Changing of transmission periodicity

**Fig 2:**
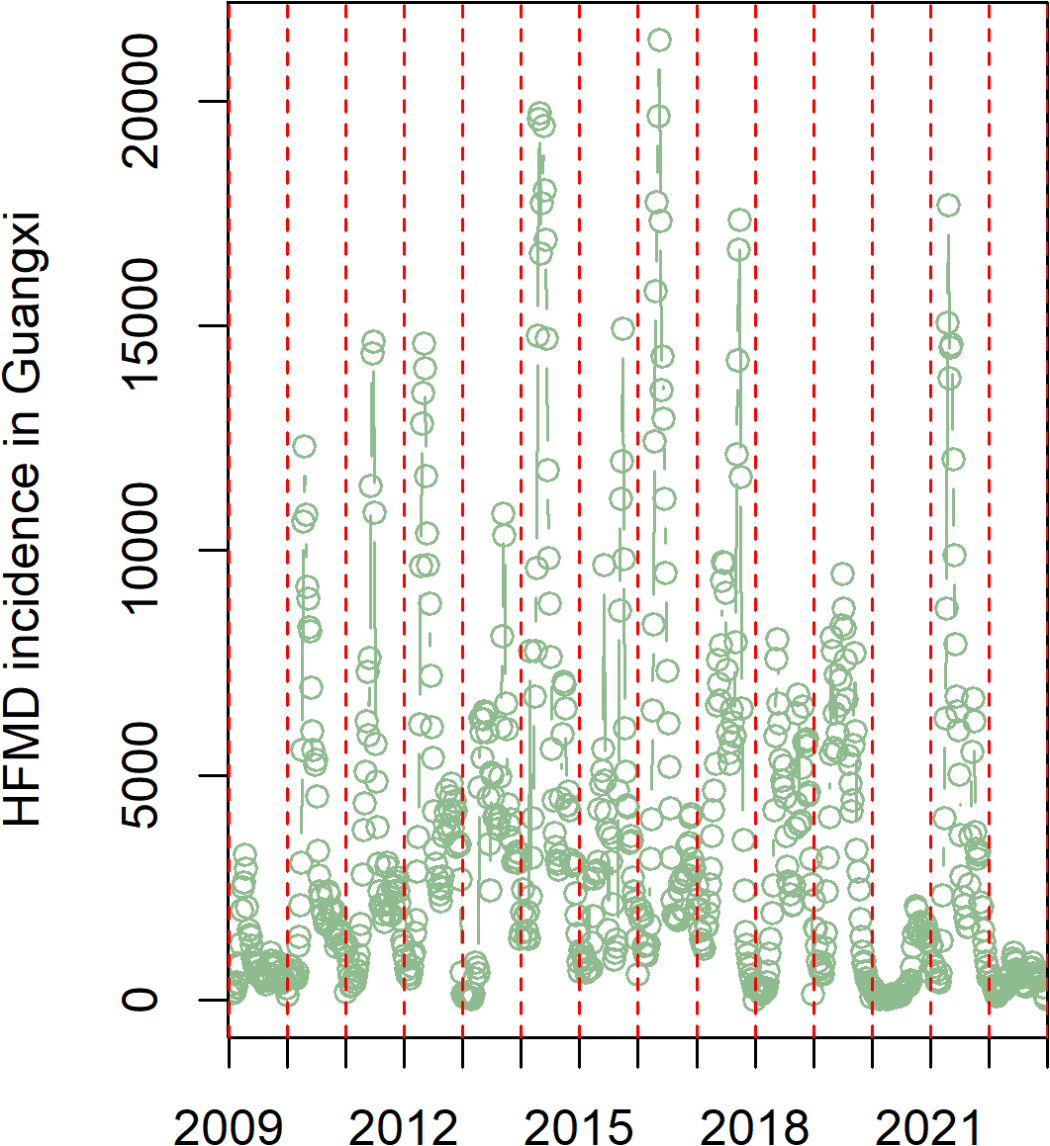
Transmission periods of weekly reported HFMD cases in Guangxi, 2009–2022. The time series reported pattern with weekly case count in Guangxi indicated an annual periodicity.

**Fig 3:**
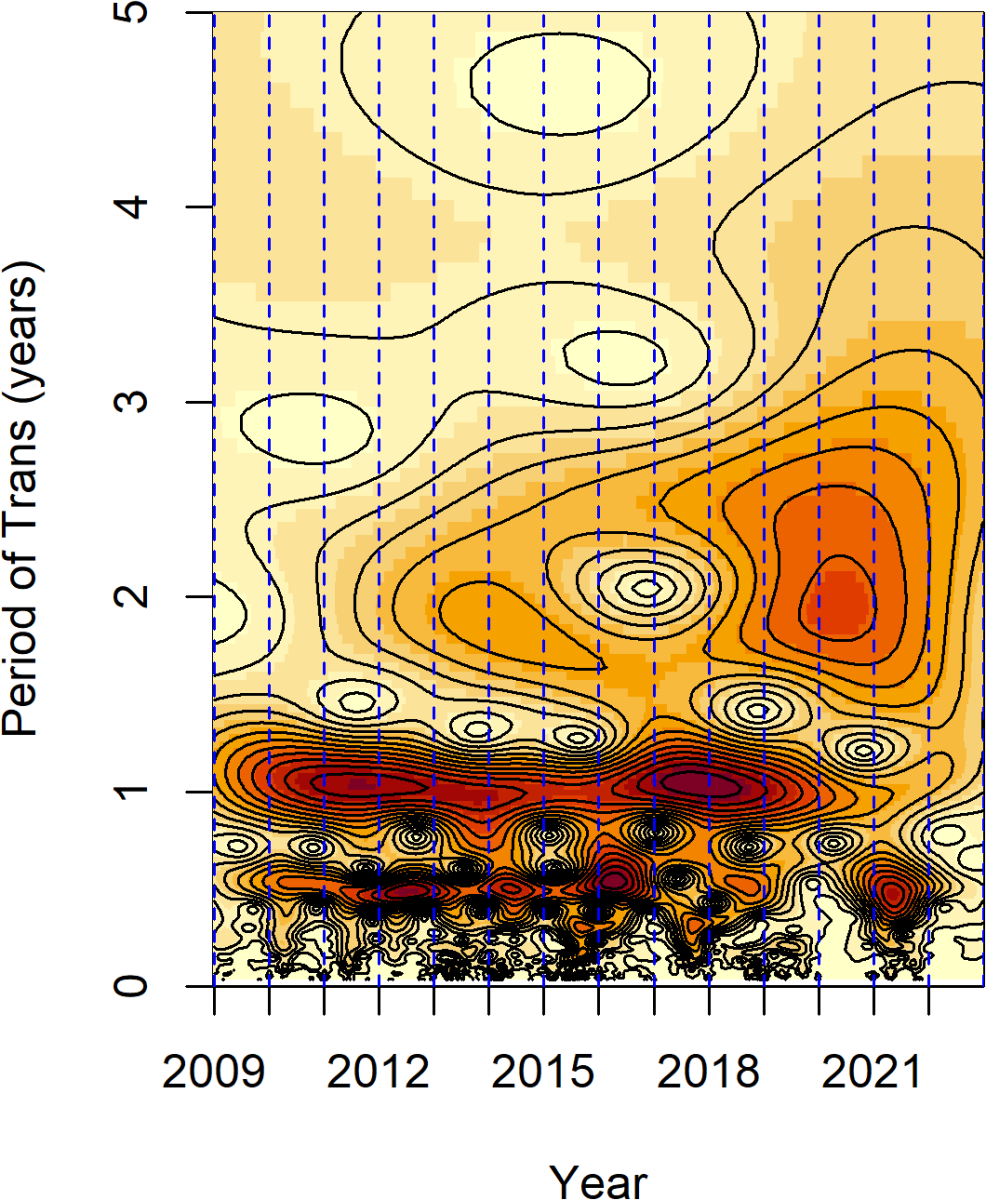
Wavelet analysis of weekly reported HFMD cases in Guangxi, 2009–2022. Wavelet power spectrum of square-root-transformed of weekly-reported cases, X-axis represents the time series (year), y-axis represent the transmission periodicity (in year). Color represents the power spectrum, strong to weak (deep-red to pale gradient).

Time-series analysis revealed that minor outbreak years were consistently followed by major outbreaks the next year (Fig 1). An increasing trend in reported cases was observed in both even- and odd-numbered years, indicating a stable one-year outbreak periodicity in the pre-vaccination period. Wavelet spectrum (WS) analysis further identified a cross-year transmission relationship in 2011–2012 and 2016–2018 (indicated by the dark-red color in Fig 2), indicating persistent transmission pattern during those years, even in the winter season. The WS analysis result also detected a half-year transmission periodicity, consistent with the time-series analysis. However, the pattern changed significantly in the post-vaccination period. The half-year transmission cycle did not persist from 2017 to 2022 and the one-year transmission cycle did not change until 2020. The transmission model was verified by the time-series data and WS results (Fig 3).

### VP1 sequencing released the evolution of major pathogens

**Fig 4:**
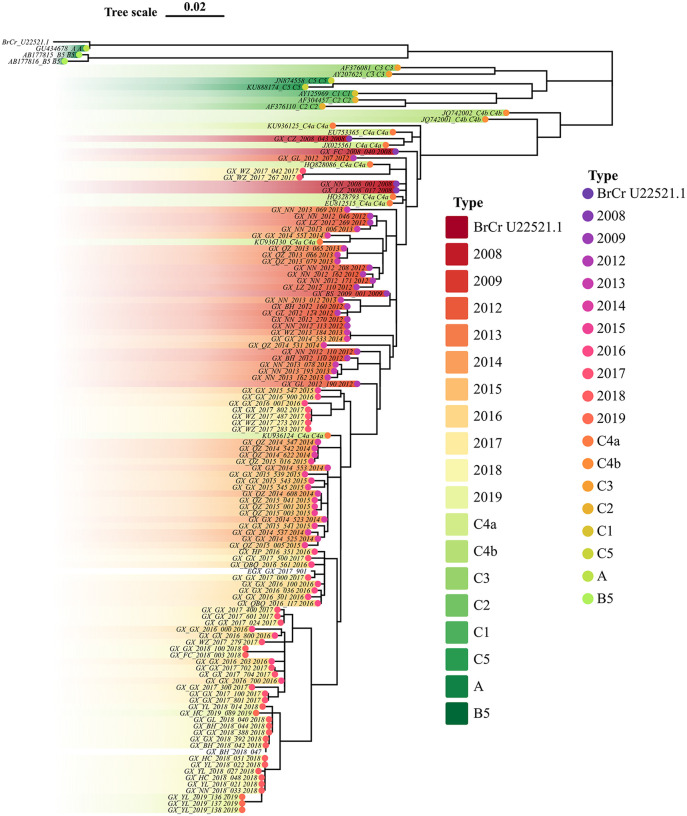
Phylogenetic trees released the evolution of EV-A71 from 2008 to 2022 in Guangxi, Southern China. The evolution of EV-A71 released that C4a linkage was responsible for all the transmission across the study period.

**Fig 5:**
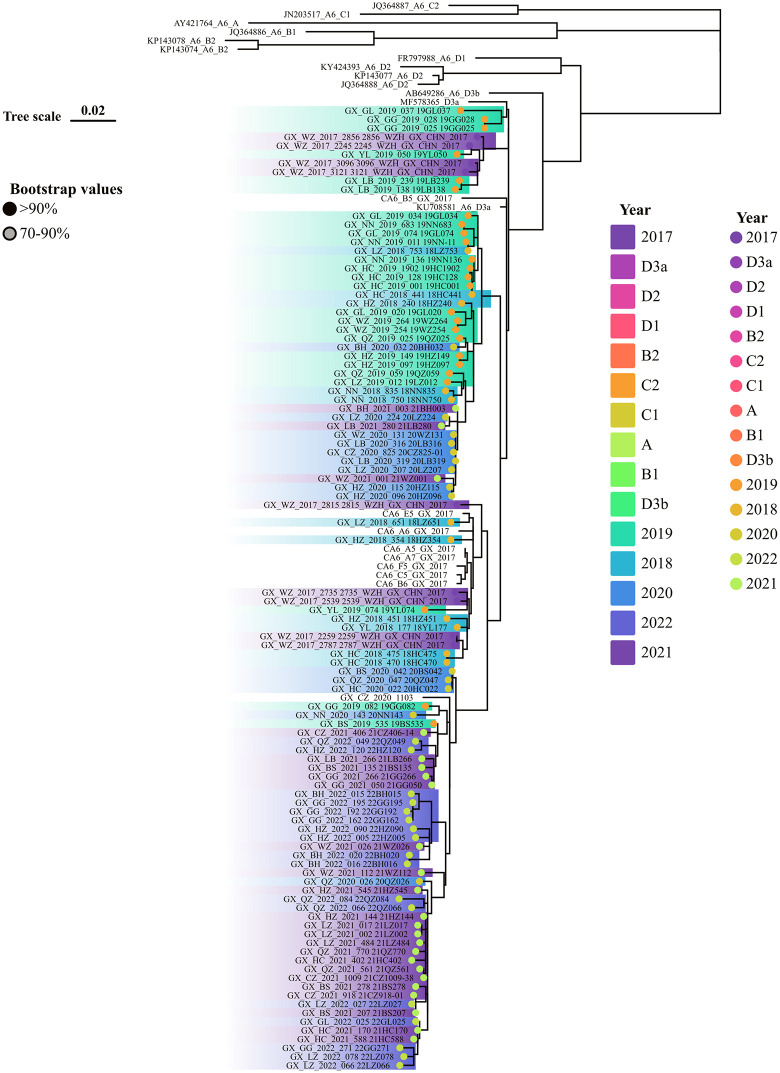
Phylogenetic trees released the evolution of CVA6 from 2008 to 2022 in Guangxi, Southern China. D3a linkage of CVA6 was the responsible strain for the transmission in the most regions.

**Fig 6:**
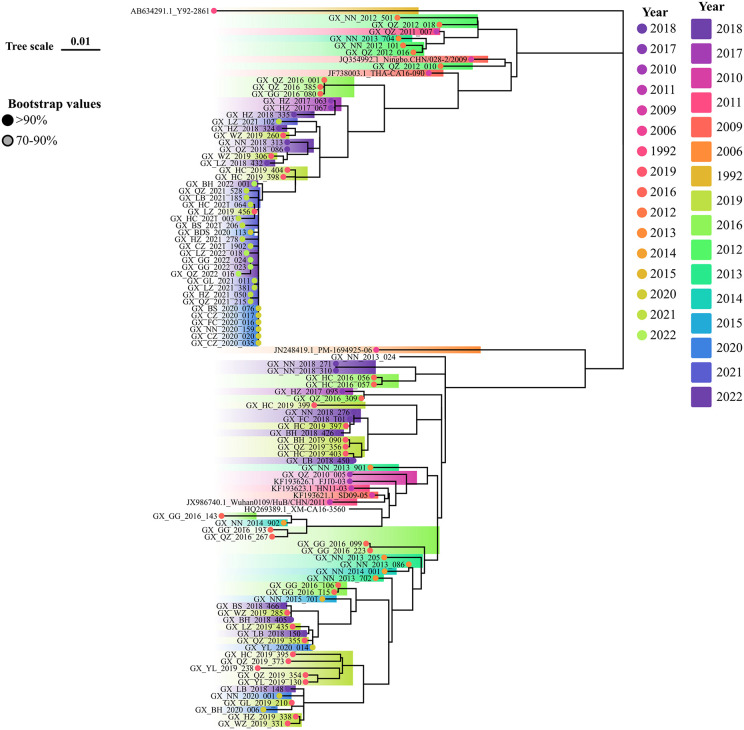
Phylogenetic trees released the evolution of CVA16 from 2008 to 2022 in Guangxi, Southern China. The co-exist two clades of CVA16 caused the transmission in different regions in Guangxi.

In our study, 17,607 EV-A71, 19,164 CVA16, 9,180 CVA6, 1,612 CVA10, and 27,942 other EVs strains were isolated between 2009 and 2022. From these, 120, 104, and 122 sequences of EV-A71, CVA16, and CVA6 strains (including references) were randomly sampled to construct phylogenetic trees to explore their evolution and transmission across Guangxi. Fig 4 highlights to two key findings regarding EV-A71 transmission in Guangxi. First, C4a was the only genotype transmitted in Guangxi. Second, the C4a strain consistently evolved to a better fitness to human. As we have seen that, cases caused by the C4a strain were generally mild, and C4a- related severe cases were on the decrease and barely detected from 2019 onward. Phylogenic analysis in Fig 5 showed that CVA6 samples collected across the study period were genetically similar and fell within the D3a primary clades. However, some cases in 2020 and 2021 had a connection with cases from previous years. Cases infected by CVA16 were grouped as two distinct clades, one originated from Ningbo city, China in 2009, the other had a close genetic relationship with strain of JN248429.1 from Shandong Province, China. CVA16-infected individuals in 2020 were closely related to those found in Baise (BS), Chongzuo (CZ), Fangchenggang (FC), and Nanning (NN) exhibiting similar genetic distances, indicating widespread circulation in these regions and indicating new transmission links without known contact (Fig 6).

### Pathogen spectrum explains changing epidemic patterns

**Fig 7:**
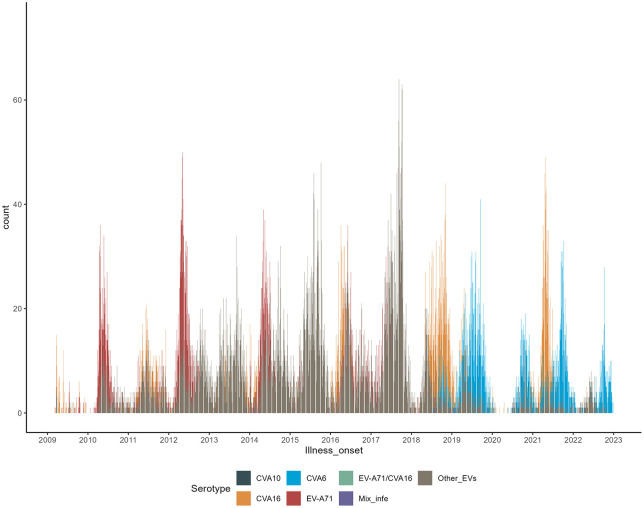
The reddish brown color represents EV-A71 serotype, the saffron-yellow color represents CVA16 serotype, the blue-dark color represents CVA10, and the light blue color indicates the CVA6 serotype. X-axis is the weeks in years; Y-axis is the cases counts of total/specific HFMD serotype. The black-blue color represents EV-A71 serotype, the saffron-yellow color represents CVA16 serotype, the brilliant blue color represents non-EV-A71-CVA16 serotype, and the light purple indicates the CVA10, and the light green color indicates the CVA6 serotype. X-axis is the weeks in years; Y-axis is the cases counts of total/specific HFMD serotype.

A total of 85.7 thousand specimens were tested for EVs from 2009 to 2022. The pathogen spectrum varied in major and minor epidemic years and in the post-vaccination period compared to the pre-vaccination period. EV-A71 was the dominant serotype in major epidemic years in the pre-vaccination period, implying that EV-A71 normally emerged in a two-year cycle, surfacing in late spring or early summer. Transmission in pre-vaccination period generally presented a two-peak pattern, with a higher, early peak in late spring or early summer and a lower, later peak in fall. The earlier peak was typically driven by EV-A71. When EV-A71 circulated with CVA16 and other EVs in spring or early summer, the prevalence reached a higher peak. In most minor epidemic years, CVA16 and other EVs were the dominant strains, emerging throughout the year and causing a minor peak. After the universal vaccination campaign, EV-A71 serotype was no longer the dominant strain (Fig 7). However, other EVs and CVA16 strains then emerged and became the majority isolates in the post-vaccination period (Table 1). The percentage of HFMD caused by CVA16 varied from 58.16%, 23.11%, 16.46%, 48.40% from 2018 to 2021, declining to 8.66% in 2022. After the CVA10 and CVA6 strains were separately classified after 2018, the percentage of CVA6 among all isolates increased from 8.67% in 2018 to 60.53% in 2022.

## Discussion

Integration of time series data and pathogen molecular data led to three key findings regarding changes in the epidemic pattern of HFMD before and after the EV-A71 vaccination campaign. First, compared to the pre-vaccination period, the post-vaccination period experienced a prolonged, lower peak that occurred in the spring season and a higher peak in the fall season. Second, the half-year transmission cycle disappeared in the post-vaccination period, while the one-year cycle did not change. Third, CVA16 and CVA6 have the potential to replace EV-A71 as the dominant strains in transmission.

In 2008, China developed an Internet-based report system to manage the increasing prevalence of HFMD. Data from this system including 7.2 million HFMD cases from 2008 to 2012 in and revealed that that HFMD in China has a seasonal transmission pattern. The highest case wave that has been observed occurred in June in Northern China and included only one major peak a year. However, HFMD cases in the Southern China appear earlier and typically have a double-peak and seasonal pattern [21]. After the launch of the EV-A71 vaccine campaign, the HFMD epidemic peak was delayed (1–2 months), but the risk of cases becoming severe increased for those infected by CVA16 and other EVs [37]. Considering this, the epidemic pattern of HFMD and predominant serotypes should continue to be closely monitored. However, the spring and fall peaks of HFMD in Southern China are still poorly understood [23]. The combination of the pathogen spectrum and the time-series surveillance data in this study revealed that differences in the pathogen spectrum have played an important role in the epidemic pattern of HFMD in Guangxi. Specifically, the epidemiological patterns were driven by the various emerging pathogens. Our time-series results showed that, in the pre-vaccination period, the EV-A71 serotype appeared earlier and surged in late spring and early summer. However, CVA16 and other enteroviruses appeared later in the year and caused fewer cases than EV-A71. Our VP1 sequencing results indicate that the fall peak was predominantly caused by CVA16 or other enterovirus serotypes, while the spring peak was caused by EV-A71 in the pre-vaccination period. We hypothesize the mechanism behind this pattern may be due to unique characteristics of these different pathogens. Some serotypes of HFMD favor the temperature and humidity in spring, while others favor the fall, which allows them to replicate and spread more easily. Additionally, previous studies have suggested that the introduction of a vaccine can induce recombination between circulating serotypes, immune escape among sub-genotype strains, and spontaneous mutations in the viral genome, which may result in changes in serotype distribution and epidemic patterns [24,42]. In the post-vaccination period, a delay and lower peak in the spring season and a higher peak in the fall season was observed. This demonstrates that the EV-A71 vaccine halted a portion of the transmission in the spring season but had no impact on reducing cases in fall. Previous studies suggest that the existence of cross-protection and partial cross-reactivity between the EV-A71 and CVA16 serotypes might have important effects on the burden of disease caused by CVA16, and other serotypes [43,44]. Without the cross-protection induced by EV-A71 transmission, CVA16 and other EVs may emerge as a predominant strain and change the epidemic pattern of HFMD in Southern China.

Various epidemic periodicities of HFMD have been observed in different regions of the world. The transmission pattern in the Asia-Pacific countries suggest a two or three-year period cycle, often in countries with high birth rates and large numbers of young children who are more susceptible [45]. Regions in Japan and Malaysia, where the birth rate is similar to Southern China, have reported a one-year transmission periodicity [46,47]. Hong Kong Special Administrative Region, Southern Taiwan, and Vietnam have had a half-year cycle of transmission [48–50], which was consistent with the pattern in Guangxi, Southern China. However, Cambodia [51], Singapore [52], and Malaysia [53] bear a three-year or two-year periodicity, exhibiting a pattern of three-year epidemic cycles. Previous studies suggested that HFMD showed a two-year epidemic pattern with a two-peak model in Guangxi [54]. However, consistent with other research, Wavelet power spectrum analysis in our study demonstrated that HFMD transmission in Guangxi presented a one-year periodicity, with some regions having a half-year transmission cycle in the pre-vaccination period [21,24]. Cities with large population sizes and high birth rates in Guangxi experience a high degree of population mobility, in which susceptibility to HFMD among young children accumulates in a short period of time. Consequently, HFMD cases are always observed in the early summer and autumn season. The half-year transmission cycle disappeared in the post-vaccination period, likely due to the universal immunization campaign of EV-A71 vaccine that has reduced cases caused by the EV-A71 serotype, which was the predominant pathogen associated with the half-year cycle.

The monovalent-inactivated EV-A71 vaccine was developed based upon VP1 sequencing from a C4 strain EV-A71 that was obtained during a 2008 outbreak in Fuyang, Anhui province, China. The vaccine efficacy was 97.4% (95% confidence interval [CI]: 92.9% to 99.0%) and provided strong protection against wide spectrum of EV-A71 sub-genotypes associated with HFMD in infants and young children [55]. VP1 contributes to the majority of neutralizing epitopes. Among the common HFMD-related EVs, EV-A71 shows high conservation in capsid proteins [56]. In this study, we demonstrated that C4 strain of EV-A71 continued to evolve after the vaccine deployment in 2016. These findings raise concerns about changes in predominant HFMD-causing EVs and emerging new disease-causing sub-genotypes. It is potential that loss of vaccine-induced immunity could lead to subsequent immune escape. Follow-up research is needed to assess whether immunity against non-vaccine sub-genotypes decline more rapidly over time.

EV-A71 is an RNA virus which contains a positive sense, single-stranded RNA genome. During the process of viral genome replication, there is no proofreading activity of RNA-dependent RNA polymerase. The instinct mechanism of RNA replication results in high mutation levels and genetic diversity of the EV-A71 viral genome. Mutants have an association with major clinical and biological impacts such as the loss of immune barriers built by previous vaccination, immune escape, vaccine breakthrough infection, and case surge. However, the scope of the data in this study did not allow us to investigate the vaccine breakthrough infection. We could not obtainEV-A71 vaccine recipient list. Correlations between mutations and the breakthrough infection could not be derived.

Analysis of pathogen detection data revealed that the proportion of CVA16 and CVA6 serotypes increased after the vaccination campaign, while the proportion of EV-A71 serotype consistently decreased. We hypothesize that CVA16 and CVA6 have the potential to replace EV-A71 as the predominant strains following vaccination against EV-A71. Similar serotype replacement has been observed in Shanghai [18], Yunnan [19], and other parts of China [57,58]. The decline of EV-A71 serotype in pathogen detection may also coincide with a reduction in cross-immunity against CVA16 and other serotypes. The current study found that a higher case-wave in fall was determined to be related to CV16, reflecting an increased transmission of these CVA16 sub-genotypes.

In conclusion, the combination time-series data and the pathogen sequencing data used in this study revealed the HFMD epidemic pattern has shifted and that a replacement of predominant strains has occurred after the vaccination campaign. Given the high-quality surveillance data, as well as the pathogen information that can be gleaned from VP1 sequencing, public health surveillance of HFMD should incorporate whole genome sequencing to ensure appropriate monitoring of serotype replacement and evaluate the necessity of multivalent vaccines to reduce the high disease burden caused by HFMD in Southern China.

Our study has limitations. First, serotypes CVA10 and CVA6 were separated from other EVs in 2018. Therefore, this study could not determine if CVA6 emerged as a predominant pathogen before 2017. Therefore, more time-series data is needed to confirm CVA6 as a leading pathogen of serotype replacement. Second, the serotype detection of EVs in this study was less precise. We only isolated and sequenced EV-A71, CVA16, and other EVs before 2017. Although CVA6 and CVA10 were added to the list of isolation and sequencing, more robust data with additional pathogen detection is required to fully assess seasonal patterns and predominant serotype replacement.
